# The Case for Using a Behavior Change Model to Design Interventions to Promote Respectful Maternal Care

**DOI:** 10.9745/GHSP-D-22-00278

**Published:** 2023-02-28

**Authors:** Nadia Diamond-Smith, Dilys Walker, Patience A. Afulani, France Donnay, Sunny (Pei Yi) Lin, Emily Peca, Mary Ellen Stanton

**Affiliations:** a Department of Epidemiology and Biostatistics, University of California, San Francisco, San Francisco, CA, USA.; b Institute for Global Health Sciences and University of California, San Francisco, San Francisco, CA, USA.; c Department of Obstetrics, Gynecology, and Reproductive Sciences, University of California, San Francisco, San Francisco, CA, USA.; d King’s College London, London, United Kingdom.; e University Research Co., LLC., Chevy Chase, MD, USA.; f Consultant, McLean, VA, USA.

## Abstract

Applying a behavior change framework to guide the design of interventions to improve respectful maternity care (RMC) could accelerate and unify the implementation and evaluation of diverse RMC interventions.

## Key Messages

- With increased evidence of the importance of promoting respectful maternity care (RMC), and more interventions being developed, implementers and intervention developers could learn from existing frameworks to guide the design of more effective interventions.
- We argue that a behavior change framework, such as the capability, opportunity, and motivation behavior model, holds value for moving the needle on RMC because respectful care relies primarily on the behavior of the provider.
- We map existing interventions to this framework to highlight opportunities for learning from existing interventions and to guide future development.

## INTRODUCTION

Respectful maternal or maternity care (RMC) refers to care provided to women at the time of birth that assures respect, trust, and communication, as well as lacking any form of abuse or neglect. RMC ensures that women giving birth are informed, supported, and feel that they have a say in their birth experience.^1^ Broadly, RMC extends beyond the event of childbirth itself to encompass a woman’s experience throughout the pregnancy, delivery, and postpartum period. A wide body of literature documents disrespect and abuse of poor women/person-centered care globally, and there is a growing need to develop interventions that improve RMC.^2^^–^^4^ Given this increased demand for interventions to promote RMC, it is important to consider interventions that are grounded in a theoretical framework, such as behavior change or implementation science theory, to clarify efforts, share lessons, and align the global community. We use the term women (and “she,” “her”) in this article to be consistent with the literature cited and as a biological variable but recognize that this does not include all people who give birth. Thus, it is inclusive of the experiences of all people who are pregnant or have given birth.

At the core of RMC is a woman’s experience, much of which is determined by the interpersonal interactions with those providing her care—in other words, a set of behaviors of the providers. These behaviors are necessarily influenced by facility characteristics and the system with which she interacts, including the culture. Implementation science frameworks have been suggested to be useful for designing behavior change interventions because such frameworks can help establish a structure to identify the targets for an intervention and design and test interventions that specifically target embedded constructs.^5^ Yet, most RMC interventions do not use implementation science or behavior change frameworks to inform intervention strategies. In fact, most maternal health interventions do not use implementation science frameworks that may help facilitate the spread of evidence-based practices.^6^ A recent scoping review of implementation science in maternal care (not specifically related to respectful care) found that only 14 of the 144 articles identified referred to a framework and that 4 of these were implementation science frameworks (self-defined by the authors as such).^7^ The most common theory used in the 14 was determinant theory (5 articles), followed by implementation science and then classic theory.

In this article, we seek to provide an example of how RMC interventions could be informed by an implementation science framework using the COM-B model (capability–opportunity–motivation that leads to behavior change) and related behavior change wheel (BCW).^8^ This is important because although the issue of RMC is increasingly recognized and featured in policy, there are gaps in terms of what works, how, and in which contexts. Viewing interventions through a behavior change lens can help intervention developers analyze interventions in a systematic way linked to a theoretical underpinning.

Viewing RMC interventions through a behavior change lens can help close the knowledge gaps in terms of what interventions work, how, and in which contexts.

## USING BEHAVIOR CHANGE FRAMEWORKS TO IMPROVE RMC INTERVENTIONS

There are a variety of theories, schemas, and approaches that are used in implementation science. Depending on the objectives in an implementation science approach, there are 6 broad categories: (1) process models (Exploration, Preparation, Implementation, Sustainment [EPIS] model; Practical, Robust Implementation and Sustainability Model [PRISM]); (2) determinant frameworks (active implementation, consolidated framework for implementation research [CFIR]), (3) implementation theories (organizational readiness); (4) implementation climate (Reach, Effectiveness, Adoption, Implementation, and Maintenance [RE-AIM], implementation outcomes); (5) classic theories (behavioral and organizational theory); and (6) evaluation frameworks.^9^ We selected a classic theory—behavior change or COM-B—because our objective was to review implementation strategies while keeping the experience of care central to the framing.

**FIGURE 1:**
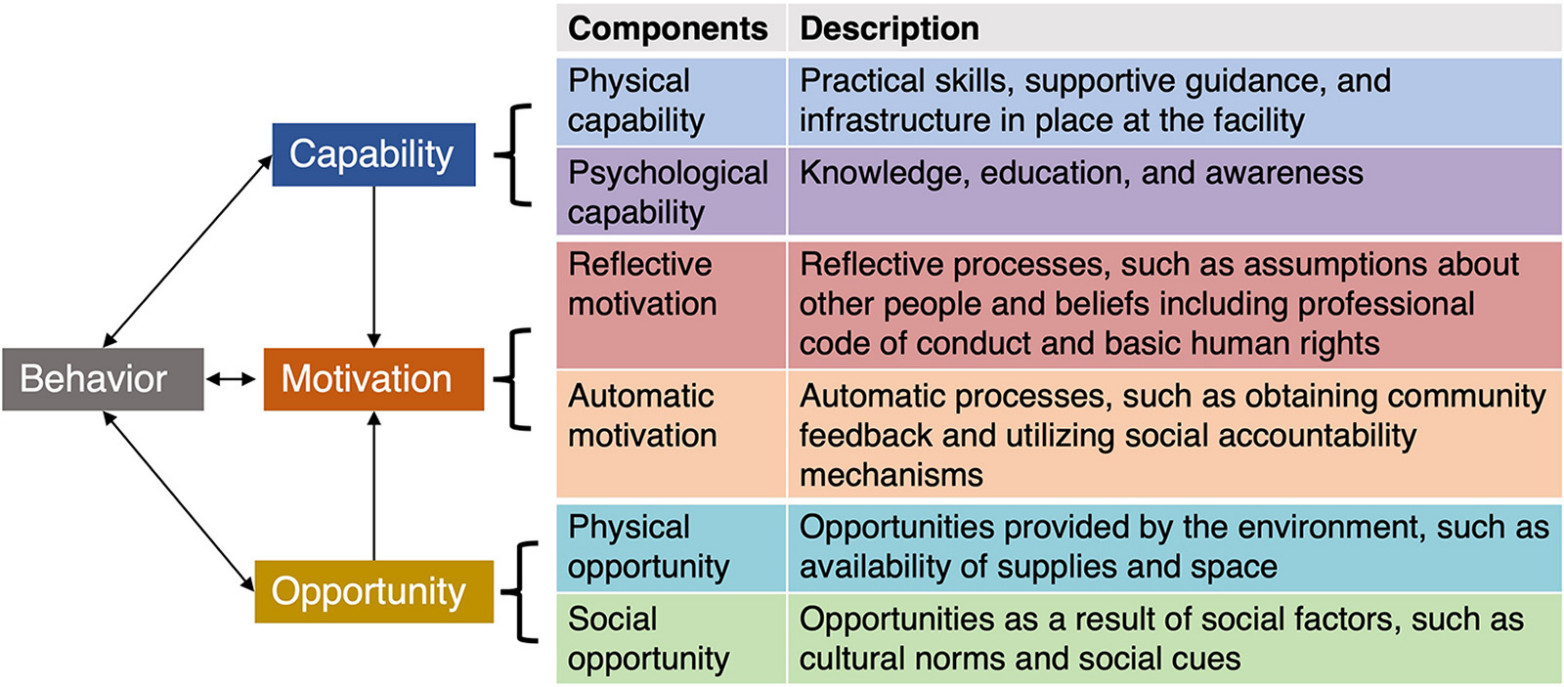
The COM-B Model With Representative RMC-Promoting Interventions^8^ Abbreviations: COM-B, Capability, Opportunity, and Motivation Behavior; RMC, respectful maternal care.

**FIGURE 2:**
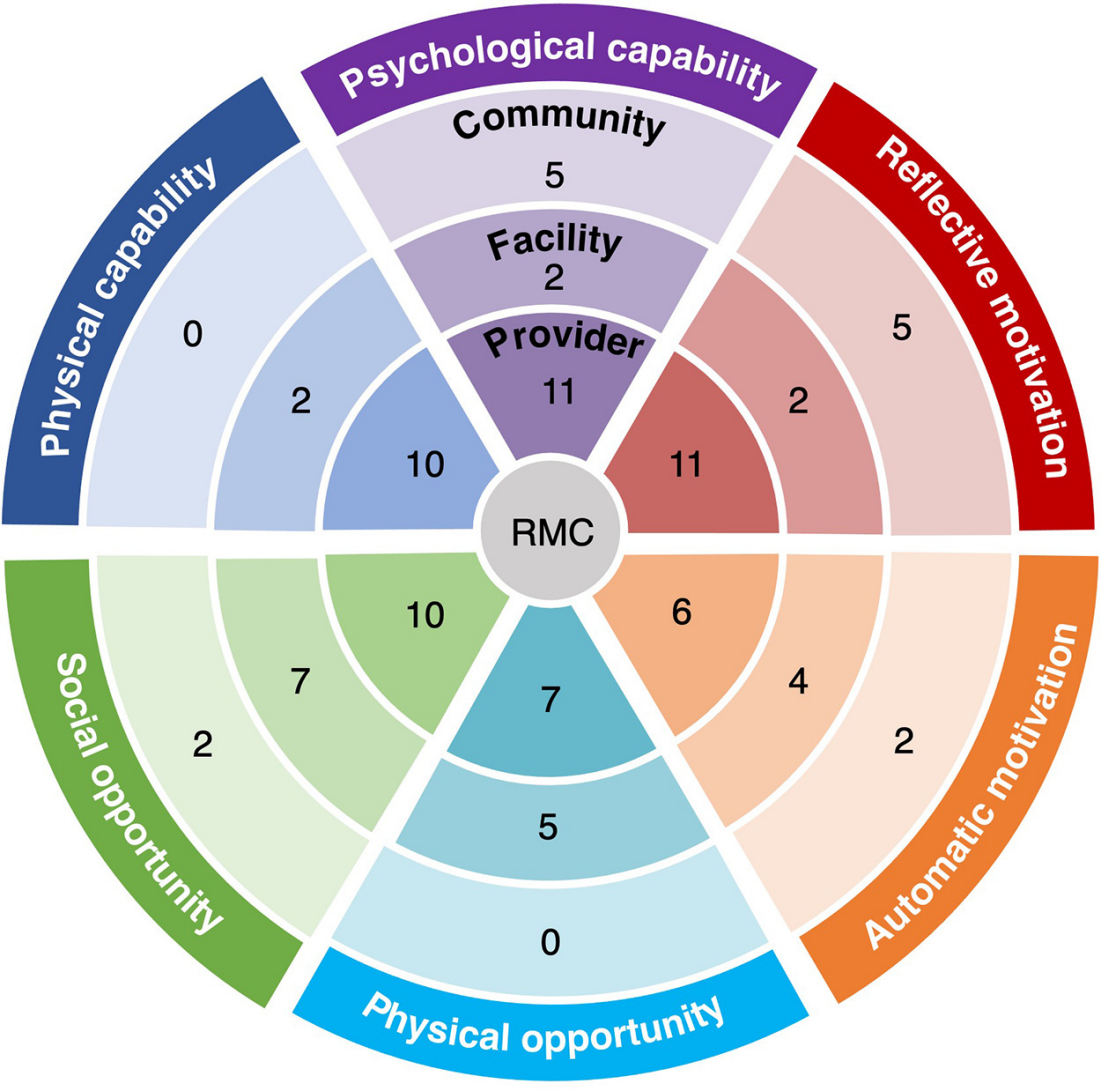
Mapping of 16 RMC-Promoting Interventions Using the Behavior Change Wheel^a^ Abbreviations: RMC, respectful maternal care. ^a^ Each intervention may have 1 or multiple approaches mapped.

The COM-B model (Figure 1) is at the center of the BCW framework (Figure 2), and together, these help identify barriers and enablers to a targeted behavior, in this case, RMC. The COM-B model posits that people need a combination of capability, opportunity, and motivation to successfully change a behavior.^8^ For example, a provider not only needs the knowledge of how to act (capability) but also requires an environment that structurally and functionally supports the behavior (opportunity) and whether there is a personal or institutional incentive or code of conduct to adopt the behavior (motivation). COM-B also emphasizes that successful individual behavior change requires drivers of change acting at multiple levels, including the individual, facility, community, and/or policy, similar to a health systems–level approach. The BCW framework helps identify whether capability, opportunity, and motivation-related factors drive a specific behavior. Once the barriers and enablers are identified in a particular context, the BCW provides guidance to frame the design of interventions by helping broaden our thinking about the multiple pathways and types of approaches that might change behavior, targeting known barriers or facilitators. It also may encourage integrated approaches that touch on multiple domains of the COM-B model acting at different levels to impact the provider-patient interaction and, ultimately, the women’s experience. Such an approach promotes commitment to the woman’s perspective as the critical measure of success, with a focus on quality as measured from the woman/patient’s perspective.

The COM-B model and BCW have been used to provide a structure for framing barriers and facilitators of various practices in both high- and low- and middle-income countries, from chlamydia testing among young people to increasing physical activity in pregnant women to integrating mental health care into primary practice.^10^^,^^11^ The COM-B model has also been used as a framework to guide intervention design for researchers working to improve health outcomes and behaviors related to an equally wide range of topics.^12^^–^^14^ Thus, COM-B and the BCW have utility as a framework not only for understanding the challenges that need to be addressed but also for designing the intervention itself. Additionally, in cases where multiple interventions already exist, the BCW can be used to map the intended pathways of previous or existing behavior change efforts. By applying this to RMC, implementers and researchers can better understand the drivers and levers needed for changing provider behavior, as well as patient and family member behavior, that will impact the woman’s perception of care.^15^^,^^16^ This analysis can also help researchers design more complex intervention packages by allowing for a better understanding of the array of evidence-based interventions and the varied pathways for impacting behaviors likely to change a woman’s experience of care. Such a mapping can also highlight gaps in the current interventions and areas ripe for innovation.

Applying the BCW to RMC can help improve understanding of factors the affect provider and patient behavior that will impact women’s perception of care.

A recent scoping review that applied the COM-B model identified only 1 article in the field of maternal care.^17^ This study, in the United Kingdom, used the COM-B model to guide the development of an intervention aimed to improve the quality and quantity of discussions between midwives and women at low risk of pregnancy complications about their choice of delivery location. The midwifery model of care is situated in a women-centered/women’s experience framework, and thus, many components of this intervention also addressed domains of RMC. The intervention was designed to impact the behavior of midwives providing care grounded in the COM-B model and evaluated their behavior.

## MAPPING RMC INTERVENTIONS TO THE COM-B MODEL AND BCW

In a recent landscaping review, we identified 43 interventions implemented across Africa promoting RMC between 2009 and 2020, of which 16 were unique.^18^ The details about the interventions (geography, content, target population, and measures) and methods of the review are published elsewhere.^18^ Briefly, we searched 3 databases, PubMed, Embase, and Web of Science, to find documentation of RMC-promoting interventions in Africa between 2009 and 2020. Our search terms were informed by the Respectful Maternity Care Charter developed by the White Ribbon Alliance.^19^ Information on intervention time period, design, scope, level, target audience, and any other characteristics were collected. Interventions that only targeted antenatal care or the postpartum period were excluded because the primary focus of the parent study was on RMC at the time of delivery. We focused on Africa to keep the scope and findings of our review comparable. The methods and analysis were conducted by the authors of this article in an iterative and collaborative process.^18^

This article leverages the same set of articles to consider if and how programs are using implementation science frameworks, especially the COM-B model, to design and evaluate RMC interventions. In this article, we aim to map what frameworks or framings were applied to these interventions and to attempt to apply the BCW to map each of the identified intervention approaches onto the COM-B model. We further identify and discuss gaps in the intervention approaches by applying this behavior change lens.

For this analysis, we excluded interventions identified through gray literature or crowdsourcing because of incomplete and inconsistent information. We included 16 published interventions in this exercise.^2^^,^^20^^–^^34^

Each publication describing the intervention was reviewed by authors SL, NDS, and DW, for reference to a behavior change or related foundational theory, framing, or framework that grounded the intervention design or strategy. After that, SL, NDS, and DW extracted information on approach(es) taken by each intervention to promote RMC and matched them to the 6 components of the COM-B model. They met to discuss pathways and reached consensus for each intervention. Figure 1 shows the COM-B model’s 6 components in 3 main domains: capability (physical and psychological), motivation (reflective and automatic), and opportunity (physical and social), and provides a brief description of example intervention approaches related to RMC that might fall within each of those. The reviewing authors met to discuss pathways for each intervention and reached consensus for mapping to the 6 components of the COM-B model and onto the BCW.

A few articles mentioned behavior change broadly (though not all). Only 1 article specifically mentioned behavior change and implementation science,^29^ and even cited the foundational COM-B article. However, the authors did not discuss COM-B in the text and provided little discussion of how implementation science or behavior change frameworks were integrated into the approach.^29^ The World Health Organization’s Quality of Care framework and the Universal Rights of Childbearing Women (more of a framing than framework) and documents related to these frameworks were the most commonly mentioned.^19^^,^^35^

From the COM-B framework (Figure 1), we expanded our focus to the BCW (Figure 2). We conceptualized the wheel with the 3 layers representing different levels of the system in which a woman interacts—with the experience of RMC at the center. The concentric circles moving outward show the different layers of the system that she interacts with and the different potential targets for interventions. In the first circle is the provider and interventions may include provider training, provider-focused activities to alleviate provider stress and burnout, or programs to shift providers’ underlying code of conduct or cultural and gender norms that might perpetuate poor RMC. The next circle represents the facility, where interventions could target structural factors, such as curtains or clean bathrooms, as well as better supervision or facility-level activities to alleviate drivers of poor RMC, such as workload. The outermost circle represents community-level interventions, such as wider awareness raising about women’s rights or social accountability approaches. This circle is where community-level cultural or gender norms-shifting also occurs. Lastly, we recognize that there are a variety of national or global policy-level interventions that play an important role in establishing guidelines and standards for provider behaviors and practice, but these were not the focus of this exercise.

Figure 2 shows the distribution of the 16 identified interventions and their focus of action. The numbers represent the number of interventions using the specific strategy. Most interventions used multiple strategies at multiple levels. Social opportunity (N=19), psychological capability (N=18), and reflective motivation (N=18) were the components that were most often represented (keep in mind that some interventions targeted more than 1 layer of the circle, and thus these N’s are greater than the total number of articles reviewed). Automatic motivation (N=12), physical opportunity (N=12), and physical capability (N=12) were less commonly a part of published RMC interventions. Of note, most interventions that we analyzed touched on several different COM-B components and generally drew from at least 2 of the bigger domains (capability, opportunity, and motivation). The Table provides details of each intervention and how we categorized each using the COM-B levels and targets.

**TABLE.** Description of Intervention Activities Mapped Onto COM-B Model Components or Domains and BCW Target Audience

Reference	Country, Level	Description	Target Audience	**COM-B Domain**					
				Capability		Opportunity		Motivation	
				Psychological	Physical	Social	Physical	Automatic	Reflective
Abuya et al.^2^	Kenya, facility	RMC training, mentorship, QI team, D&A monitoring, improved infrastructure, maternity open days, community workshops and counseling, mediation/alternative dispute resolution	Provider	X	X	X	X	X	X
			Facility			X			X
			Community	X				X	X
Afulani et al.^20^	Ghana, district	RMC and emergency obstetric and neonatal care training	Provider	X	X				X
Apolot et al.^21^	Ethiopia, subcounty	Community score cards, meetings, and health education	Community					X	X
Asefa et al.^22^	Ethiopia, facility	RMC workshop, visual aids, RMC standard checklist, QI supportive supervision visits	Provider	X	X	X	X		X
Fujita et al.^23^	Benin, facility	Mother’s childbirth class and infrastructure improvements	Provider		X			X	X
			Facility			X	X	X	
			Community	X		X			X
Giessler et al.^24^	Kenya, facility	Model for improvement: clear aim, monitor progress with repeated measurements, and test ideas	Provider			X			
Habib et al.^25^	Ghana (Multiple), region	“RMC for healthcare workers: tackling D&A during facility-based childbirth project” training package	Provider	X	X	X	X		X
Kongnyuy et al.^26^	Malawi (Multiple), district	Criteria-based audit	Provider		X	X			
			Facility	X	X	X			
			Community	X		X			
Kujawski et al.^27^	Tanzania, region	Maternity open days, workshops, infrastructure improvements, counseling staff, observation/monitoring, provider dispute resolution training	Provider	X		X	X	X	X
			Facility			X	X	X	
			Community	X					
Mihret et al.^28^	Ethiopia, facility	RMC training, RMC planning and monitoring, facilitating patient/family’s involvement, teamwork and communication, infrastructure improvements, guidelines for nurses	Provider	X		X	X	X	X
			Facility			X	X	X	
Oosthuizen et al.^29^	South Africa, district	Essential childbirth and newborn care training (RMC values embedded in all activities)	Provider	X	X	X	X	X	X
			Facility	X	X	X	X	X	X
Ratcliffe et al.^30^	Tanzania, facility	RMC workshops and open birth days	Provider	X				X	X
			Community	X					X
Umbeli et al.^31^	Sudan, facility	Provider training on patient’s communication during labor	Provider	X					
Wilson-Mitchell et al.^32^	Tanzania, zone	Intellectual Partnership Model to teach RMC, lecture, pair and share, role play	Provider	X	X	X		X	
Webber et al.^33^	Tanzania, district	Interactive HCW training addresses provider roles, gender	Provider	X	X	X			X
Zethof et al.^34^	Malawi, facility	Promotion of informed consent through standardized checklists, guide, and HCW training	Provider	X	X		X		X
			Facility			X	X		
			Community						X

Abbreviations: BCW, behavior change wheel; COM-B, capability–opportunity–motivation that leads to behavior change; D&A, disrespect and abuse; HCW, health care workers; QI, quality improvement; RMC, respectful maternal care.

## IMPROVING WOMEN-CENTERED CARE WITH MORE EFFECTIVE RMC INTERVENTIONS

It is time to move beyond building the evidence base to describe and document the disrespect and abuse that women experience during childbirth. Developing and testing effective interventions to improve RMC is essential. Behavior change frameworks, such as the COM-B model, provide a useful analytic tool to guide implementers in designing interventions aiming to improve RMC. We outline the types of intervention targets that fit at various levels within the COM-B framework and BCW (provider, facility, and community) and address the 6 different COM-B components. Other behavior change, health system strengthening, or implementation frameworks could also be applied to the design and evaluation of RMC interventions. However, fundamentally, in this article, we argue that drawing from established frameworks founded in behavior change theory keeps the woman at the center of care and could accelerate understanding and advancement in the field.

We argue that drawing from established frameworks founded in behavior change theory keeps the woman at the center of care.

Other scholars have suggested a health systems framework to anchor interventions to promote RMC. However, such an approach, for example, the WHO Health Systems Framework for Action, instead of focusing on measures of a person’s experience and perception of care, focuses on health system strength or functioning and risks losing sight of the woman’s experience of care.^36^ A recent article by Cometto et al. used a health workforce lens to outline policy levers to promote what they called compassionate, respectful care, which focused on health workforce governance and management.^37^ Cometto et al. looked at interventions to promote RMC, as well as compassionate, respectful care for other health outcomes (e.g., HIV). While their framework is focused on policy levers and organized with a different underpinned theory, it highlights many of the same strategies that we identified in our analysis, such as accountability mechanisms, supervision, training, and incentives for providers, and lays out how these approaches can enable and reinforce each other at different levels of the health workforce system. Our approach is similar and aims to keep the woman and her experience at the center.

As has been found elsewhere in reviews of maternity-related interventions, few studies describing interventions on maternal care used (or discussed in their publications) a framework to guide their design.^5^^,^^7^ When focusing on RMC interventions in Africa, we find that non-implementation science frameworks are used most (quality of care or human rights). In contrast, implementation science frameworks, such as COM-B and the BCW, provide structured guidance to implementers on how to think about where to intervene and how, and, ideally, help broaden their intervention strategies to increase the chances of behavior change. Hearteningly, most of the interventions that we analyzed did implicitly capture multiple COM-B model domains, suggesting a logical connection to behavior change. We recommend that implementers working in this field consider mapping their interventions onto behavior change frameworks such as the BCW, with COM-B at the center, to ensure that they are leveraging best practices to promote behavior change. By pulling from all domains of the COM-B models within interventions, implementers may be able to design interventions that are most likely to have an impact on the desired outcome (RMC), which must measure success based on experience of care measures. Furthermore, this approach provides a structured approach to guide monitoring and evaluation activities, for example, measuring knowledge (capability), as well as norms (opportunity) and motivation (outcomes across COM-B domains).

Our mapping exercise highlighted some specific components of COM-B that were most commonly the target for interventions. Educating providers and community members about RMC (psychological capability) and addressing beliefs and norms (reflective motivation and social opportunity) are important to raising awareness; thus, it makes sense that these were core to many interventions. Changing the physical structure of facilities or the staffing and guidelines in those facilities (physical opportunity and capability) requires more buy-in from various stakeholders and resources, which may account for their less frequent representation. Automatic motivation interventions, such as social accountability and community engagement, may also be more challenging to implement and are a somewhat newer approach, so they might become more common with time. An intervention addressing only 1 or 2 domains of the COM-B model may result in limited sustained behavior change. For example, if we provide education to providers about RMC (capability) but do not change the physical opportunity to act on that knowledge (give them more time with women or more private rooms to communicate clearly to women), then they will be unable to act upon the knowledge that they have gained. The finding that the vast majority focus on the provider level is understandable, given that much of respectful care ultimately lies in the interaction between a provider and the woman. However, supporting providers to enable them to act upon the knowledge gained is essential and requires activities at multiple levels. For example, changing norms, such as around accepting women’s agency to advocate for the care she desires and other gender norms, at the community level and engaging supervisors at the facility level may create the enabling environment needed for lasting provider behavior change.

We add to the literature by exploring how an implementation science framework, in this case, the COM-B model, could be applied to interventions aiming to improve RMC. By carefully examining existing interventions and mapping them onto the BCW, we provide practical and actionable insights to help guide, or at least model, the applicability of COM-B and the BCW to future intervention design, adaptation, or scale-up. This approach will also help structure evaluations and measures of impact for complex interventions.

We provide practical and actionable insights to help guide the applicability of COM-B and the BCW to future intervention design, adaptation, or scale-up.

### Limitations

This exercise does have limitations. We were limited to interventions related to RMC in Africa published from 2010 to 2021. Thus, in addition to missing older interventions, it is also possible that interventions in other regions of the world use different approaches, leading to different insights. Our goal was not to be exhaustive but rather to conduct an exercise to see how a subset of existing interventions could be reframed with this implementation science model and describe the potential value of doing so. Another key limitation is that we had to rely on the information in published articles about the intervention design and make decisions about which COM-B domain the intervention components fit under. Due to journal restrictions on word counts, in some cases, information was limited, which may have restricted our ability to assign domains. Fundamentally, we see this as an exercise in applying this framework and model to RMC; others with different expertise may map these interventions slightly differently. Our goal in this article is to show that this framework could have utility to future designers and implementers of RMC interventions. Equally important is to identify a framework that is a valuable tool with unifying principles to evaluate intervention impact. As with any qualitative classification like this, the team’s perspectives likely introduced some biases into the interpretation. Our team comprises clinicians and epidemiologist researchers in the RMC community, many of whom have decades of experience designing, evaluating, and funding projects aimed to improve RMC. We also consulted a group of RMC experts convened through the U.S. Agency for International Development–funded Health Evaluation and Applied Research Development project and discussed our interpretations and analysis. It is also important to note that this analysis related to what implementers are doing or have done, not what the evidence shows about which types of interventions may be most effective. In fact, very little information on effectiveness was available. However, by designing interventions with an established theoretical framework in mind (the COM-B model), we hypothesize that developed interventions are more easily measured and more likely to be effective long term. Finally, in this analysis, we did not consider policy-level interventions to promote RMC, which are also important for ultimately leading to behavior change and should be the focus of other studies or could be layered onto this model.

## CONCLUSIONS

Leveraging existing frameworks to promote behavior change can help facilitate action toward improving RMC in Africa and globally. By applying a behavior change framework, such as the COM-B model to RMC interventions, implementers have a tool to help them frame their approaches to target behavior change effectively. The model helps tease out the factors contributing to the complexity of provider/patient interactions and encourages implementers to consider a multidomain approach that cuts across motivation, opportunity, and capability. The framework also provides an approach to guide indicator selection and monitoring for the evaluation of impact.

## Notes

**Peer Reviewed**

**First published online:** January 25, 2023.

## References

[1] Bowser D Hill K. *Exploring Evidence for Disrespect and Abuse in Facility-Based Childbirth: Report of a Landscape Analysis*. Harvard School of Public Health/University Research Co., LLC; 2010. Acessed January 12, 2023. https://cdn2.sph.harvard.edu/wp-content/uploads/sites/32/2014/05/Exploring-Evidence-RMC_Bowser_rep_2010.pdf

[2] Abuya T Ndwiga C Ritter J. The effect of a multi-component intervention on disrespect and abuse during childbirth in Kenya. BMC Pregnancy Childbirth. 2015;15(1):224. 10.1186/s12884-015-0645-6. 26394616 PMC4580125

[3] Bohren MA Vogel JP Hunter EC. The mistreatment of women during childbirth in health facilities globally: a mixed-methods systematic review. PLoS Med. 2015;12(6):e1001847. 10.1371/journal.pmed.1001847. 26126110 PMC4488322

[4] Warren CE Njue R Ndwiga C Abuya T. Manifestations and drivers of mistreatment of women during childbirth in Kenya: implications for measurement and developing interventions. BMC Pregnancy Childbirth. 2017;17(1):102. 10.1186/s12884-017-1288-6. 28351350 PMC5371243

[5] Eccles M Grimshaw J Walker A Johnston M Pitts N. Changing the behavior of healthcare professionals: the use of theory in promoting the uptake of research findings. J Clin Epidemiol. 2005;58(2):107–112. 10.1016/j.jclinepi.2004.09.002. 15680740

[6] Bauer MS Damschroder L Hagedorn H Smith J Kilbourne AM. An introduction to implementation science for the non-specialist. BMC Psychol. 2015;3(1):32. 10.1186/s40359-015-0089-9. 26376626 PMC4573926

[7] Dadich A Piper A Coates D. Implementation science in maternity care: a scoping review. Implement Sci. 2021;16(1):16. 10.1186/s13012-021-01083-6. 33541371 PMC7860184

[8] Michie S van Stralen MM West R. The behaviour change wheel: a new method for characterising and designing behaviour change interventions. Implement Sci. 2011;6(1):42. 10.1186/1748-5908-6-42. 21513547 PMC3096582

[9] Nilsen P. Making sense of implementation theories, models and frameworks. Implement Sci. 2015;10:53. 10.1186/s13012-015-0242-0. 25895742 PMC4406164

[10] Flannery C McHugh S Anaba AE. Enablers and barriers to physical activity in overweight and obese pregnant women: an analysis informed by the theoretical domains framework and COM-B model. BMC Pregnancy Childbirth. 2018;18(1):178. 10.1186/s12884-018-1816-z. 29783933 PMC5963099

[11] McDonagh LK Saunders JM Cassell J. Application of the COM-B model to barriers and facilitators to chlamydia testing in general practice for young people and primary care practitioners: a systematic review. Implement Sci. 2018;13(1):130. 10.1186/s13012-018-0821-y. 30348165 PMC6196559

[12] Barker F Atkins L de Lusignan S. Applying the COM-B behaviour model and behaviour change wheel to develop an intervention to improve hearing-aid use in adult auditory rehabilitation. Int J Audiol. 2016;55(Suppl 3):S90–S98. 10.3109/14992027.2015.1120894. 27420547

[13] Handley MA Harleman E Gonzalez-Mendez E. Applying the COM-B model to creation of an IT-enabled health coaching and resource linkage program for low-income Latina moms with recent gestational diabetes: the STAR MAMA program. Implement Sci. 2015;11(1):73. 10.1186/s13012-016-0426-2. 27193580 PMC4870786

[14] Herber OR Atkins L Störk S Wilm S. Enhancing self-care adherence in patients with heart failure: a study protocol for developing a theory-based behaviour change intervention using the COM-B behaviour model (ACHIEVE study). BMJ Open. 2018;8(9):e025907. 10.1136/bmjopen-2018-025907. 30206096 PMC6144404

[15] Atkins L Sallis A Chadborn T. Reducing catheter-associated urinary tract infections: a systematic review of barriers and facilitators and strategic behavioural analysis of interventions. Implement Sci. 2020;15(1):44. 10.1186/s13012-020-01001-2. 32624002 PMC7336619

[16] Staniford LJ Schmidtke KA. A systematic review of hand-hygiene and environmental-disinfection interventions in settings with children. BMC Public Health. 2020;20(1):195. 10.1186/s12889-020-8301-0. 32028932 PMC7006391

[17] Henshall C Taylor B Goodwin L Farre A Jones ME Kenyon S. Improving the quality and content of midwives’ discussions with low-risk women about their options for place of birth: co-production and evaluation of an intervention package. Midwifery. 2018;59:118–126. 10.1016/j.midw.2018.01.016. 29421641

[18] Diamond-Smith N Lin S Peca E Walker D. A landscaping review of interventions to promote respectful maternal care: opportunities to advance innovation and accountability. Midwifery. 2022;115:103488. 10.1016/j.midw.2022.103488. 36191382

[19] The White Ribbon Alliance (WRA). *The Universal Rights of Women & Newborns*. WRA; 2020. Accessed January 13, 2023. https://whiteribbonalliance.org/wp-content/uploads/2022/05/WRA_RMC_Charter_FINAL.pdf

[20] Afulani PA Aborigo RA Walker D Moyer CA Cohen S Williams J. Can an integrated obstetric emergency simulation training improve respectful maternity care? Results from a pilot study in Ghana. Birth. 2019;46(3):523–532. 10.1111/birt.12418. 30680785

[21] Apolot RR Tetui M Nyachwo EB. Maternal health challenges experienced by adolescents; could community score cards address them? A case study of Kibuku District– Uganda. Int J Equity Health. 2020;19(1):191. 10.1186/s12939-020-01267-4. 33131497 PMC7604956

[22] Asefa A Morgan A Bohren MA Kermode M. Lessons learned through respectful maternity care training and its implementation in Ethiopia: an interventional mixed methods study. Reprod Health. 2020;17(1):103. 10.1186/s12978-020-00953-4. 32615999 PMC7331171

[23] Fujita N Perrin XR Vodounon JA. Humanised care and a change in practice in a hospital in Benin. Midwifery. 2012;28(4):481–488. 10.1016/j.midw.2011.07.003. 21924533

[24] Giessler K Seefeld A Montagu D. Perspectives on implementing a quality improvement collaborative to improve person-centered care for maternal and reproductive health in Kenya. Int J Qual Health Care. 2020;32(10):671–676. 10.1093/intqhc/mzaa130. 33057658 PMC7737155

[25] Habib HH Torpey K Maya ET Ankomah A. Promoting respectful maternity care for adolescents in Ghana: a quasi-experimental study protocol. Reprod Health. 2020;17(1):129. 10.1186/s12978-020-00977-w. 32831100 PMC7444244

[26] Kongnyuy EJ Mlava G van den Broek N. Criteria-based audit to improve women-friendly care in maternity units in Malawi. J Obstet Gynaecol Res. 2009;35(3):483–489. 10.1111/j.1447-0756.2008.00990.x. 19527387

[27] Kujawski SA Freedman LP Ramsey K. Community and health system intervention to reduce disrespect and abuse during childbirth in Tanga Region, Tanzania: a comparative before-and-after study. PLoS Med. 2017;14(7):e1002341. 10.1371/journal.pmed.1002341. 28700587 PMC5507413

[28] Mihret H Atnafu A Gebremedhin T Dellie E. Reducing disrespect and abuse of women during antenatal care and delivery services at Injibara General Hospital, Northwest Ethiopia: a pre–post interventional study. Int J Womens Health. 2020;12:835–847. 10.2147/IJWH.S273468. 33116933 PMC7568622

[29] Oosthuizen SJ Bergh A-M Grimbeek J Pattinson RC. Midwife-led obstetric units working ‘CLEVER’: improving perinatal outcome indicators in a South African health district. S Afr Med J. 2019;109(2):95–101. 10.7196/SAMJ.2019.v109i2.13429. 30834859

[30] Ratcliffe HL Sando D Lyatuu GW. Mitigating disrespect and abuse during childbirth in Tanzania: an exploratory study of the effects of two facility-based interventions in a large public hospital. Reprod Health. 2016;13(1):79. 10.1186/s12978-016-0187-z. 27424608 PMC4948096

[31] Umbeli T Murwan IO Kunna A Ismail S Sulman MM Elmahgoub A. Impact of health care provider’s training on patients’ communication during labor at Omdurman Maternity Hospital, Sudan 2011. Sudan J Med Sci. 2014;9(4):211–216.

[32] Wilson-Mitchell K Robinson J Sharpe M. Teaching respectful maternity care using an intellectual partnership model in Tanzania. Midwifery. 2018;60:27–29. 10.1016/j.midw.2018.01.019. 29477962

[33] Webber G Chirangi B Magatti N. Promoting respectful maternity care in rural Tanzania: nurses’ experiences of the “Health Workers for Change” program. BMC Health Serv Res. 2018;18(1):658. 10.1186/s12913-018-3463-5. 30134890 PMC6106895

[34] Zethof S Bakker W Nansongole F Kilowe K van Roosmalen J van den Akker T. Pre-post implementation survey of a multicomponent intervention to improve informed consent for caesarean section in Southern Malawi. BMJ Open. 2020;10(1):e030665. 10.1136/bmjopen-2019-030665. 31911511 PMC6955547

[35] Tuncalp Were WM MacLennan C. Quality of care for pregnant women and newborns-the WHO vision. BJOG. 2015;122(8):1045–1049. 10.1111/1471-0528.13451. 25929823 PMC5029576

[36] World Health Organization (WHO). *Everybody’s Business—Strengthening Health Systems to Improve Health Outcomes: WHO’s Framework for Action*. WHO; 2007. Accessed January 12, 2023. https://apps.who.int/iris/handle/10665/43918

[37] Cometto G Assegid S Abiyu G. Health workforce governance for compassionate and respectful care: a framework for research, policy and practice. BMJ Glob Health. 2022;7(3):e008007. 10.1136/bmjgh-2021-008007. 35361661 PMC8971763

